# Association between malocclusion, caries and oral hygiene in children 6 to 12 years old resident in suburban Nigeria

**DOI:** 10.1186/s12903-019-0959-2

**Published:** 2019-11-27

**Authors:** Kikelomo Adebanke Kolawole, Morenike Oluwatoyin Folayan

**Affiliations:** Department of Child Dental Health Faculty of Dentistry, Obafemi Awolowo University, Ile-Ife, 220005 Nigeria

**Keywords:** Malocclusion, Caries, Gingivitis, Periodontal health

## Abstract

**Background:**

There are conflicting opinions about the contribution of malocclusions to the development of dental caries and periodontal disease. This study’s aim was to determine the association between specific malocclusion traits, caries, oral hygiene and periodontal health for children 6 to 12 years old.

**Methods:**

The study was a household survey. The presence of malocclusion traits was assessed in 495 participants. The caries status and severity were assessed with the decayed, missing, and filled teeth (dmft/DMFT) index and the pulpal involvement, ulceration, fistula and abscess (pufa/PUFA) index. The Simplified Oral Hygiene Index (OHI-S) and Gingival Index (GI) were used to assess periodontal health. The association between malocclusion traits, the presence of caries, poor oral hygiene, and poor gingival health were determined with chi square and logistic regression analyses. Statistical significance was inferred at *p* < 0.05.

**Results:**

Seventy-four (14.9%) study participants had caries, with mean (SD) dmft/DMFT scores of 0.27 (0.82) and 0.07 (0.39), respectively, and mean (SD) pufa/PUFA index scores of 0.09 (0.43) and 0.02 (0.20), respectively. The mean (SD) OHI-S score was 1.56 (0.74) and mean (SD) GI score was 0.90 (0.43). Dental Aesthetic Index scores ranged from 13 to 48 with a mean (SD) score of 20.7 (4.57). Significantly greater proportions of participants with crowding (*p* = 0.026) and buccal crossbite (*p* = 0.009) had caries. Significantly more children with increased overjet (*p* = 0.003) and anterior open bite (*p* = 0.008) had moderate to severe gingivitis. Poor oral hygiene (OR: 1.83; CI: 1.05–3.18 *p* = 0.033), crowding (OR: 1.97; CI: 1.01–3.49; *p* = 0.021) and buccal crossbite (OR: 6.57; CI: 1.51–28.51 *p* = 0.012) significantly increased the odds of having caries. Poor oral hygiene (*p* < 0.001), increased overjet (*p* = 0.003), and anterior open bite (*p* = 0.014) were the only significant traits associated with gingivitis.

**Conclusions:**

Crowding and buccal cross bite were associated with caries, whereas increased overjet and anterior open bite were associated with gingivitis. These findings justify the recommendation of orthodontic treatment to improve oral health.

## Background

Untreated malocclusion impacts negatively on the quality of life; severe malocclusion often is associated with functional limitation, pain, and social disability that affects the emotional and social well-being of young male and female adolescents [1, 2]. The psychological impact of malocclusion may be strong because of the aesthetic value of the face and smile [3]. Humans make social contacts through the face and smile, and perceptions or distortions of these media of social contact affect self-image and self-esteem negatively [4]. The negative impact of malocclusion on oral health quality of life starts to be perceived when children are 11 to 14 years, the age when they undergo major life changes, and the impact worsens as they grow older [5].

Evidence on the contribution of malocclusions to the development of dental caries and periodontal health is conflicting. Feldenes et al. [6] found that handicapping malocclusion, maxillary irregularity, and abnormal molar relationship were associated with the occurrence and severity of dental caries. However, Szyszka-Sommerfeld and Buczkowska-Radlińska [7] reported that the influence of malocclusion on the development of dental caries and periodontal disease was minimal, and Vellappally et al. [8] found no correlation between the severity of malocclusion and dental caries among adolescents. Some researchers have reported that crowding causes improper contacts between neighboring teeth, making effective oral hygiene difficult. The difficulty with cleaning of crowded teeth increases plaque accumulation and predisposes to the development of dental caries and periodontal disease [6, 9].

Helm and Petersen [10] controlled for the effect of sex and social group, and found that crowding, extreme maxillary overjet, and cross-bite increase the risk for periodontal disease in the maxilla. Ngom et al. [11] also reported that malocclusion was a risk factor for periodontal disease. A prior study conducted in this study environment found a weak relationship between malocclusion, lip competence and gingival health [12]. A cause and effect relationship could not be established between malocclusion and gingivitis, however.

van Gastel et al. [13] reviewed the literature on the impact of orthodontic appliances used for the treatment of malocclusion on periodontal health. They found contradictory findings in the review, which they attributed partly due to the selection of materials for review and differences in the research methods employed. The literature, however, is consistent in the view that untreated malocclusion worsens the oral health-related quality of life [5]. Untreated malocclusion increases the risk for caries [6], poor oral hygiene and poor gingival health [9], thereby causing pain and functional limitation. As with malocclusion [14], the quality of life is affected by caries [15], poor oral hygiene [16], and poor gingival health [17].

For this study, we examined the association between malocclusion traits, caries, oral hygiene and gingival health for children 6 to 12 years old. It was important to identify the further possible impact of malocclusion on the oral health of children and adolescents with mixed dentition.

## Methods

This is a secondary analysis of data generated to determine the association between oral habits, caries [18], and periodontal health [19]. The study was a household survey of children resident in Ife Central Local Government Area of Osun State, a suburban area in Nigeria. The data were collected in the months of August and September 2013.

The study methodology has been extensively described by Folayan et al. [20] and Kolawole et al. [18]. Children 6 months to 12 years of age whose parents consented to their participation in study were recruited. Child’s sex was defined as the biological sex; age was defined as the age at last birthday. For children less than a year old, the age was defined as the number of months after birth.

**Fig. 1:**
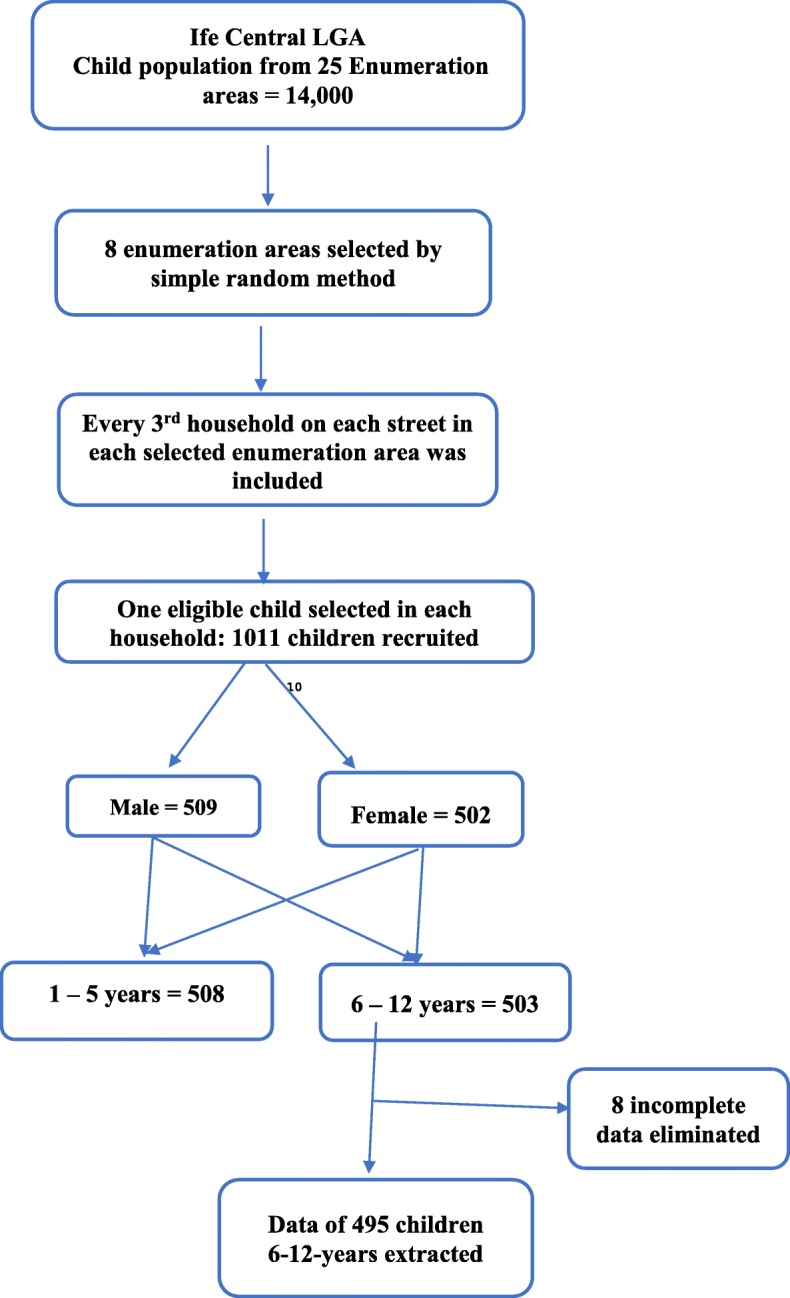
Flowchart of sampling conducted for the primary study and the data extracted for this study

Sampling was done with a multi-stage technique (Fig. 1), that involved random selection of enumeration areas within the Local Government Area; selection of every third household on each street; identification of eligible individuals within households; and selection of respondents for interview and clinical examination. Only one child per household was selected for study participation. A structured questionnaire was used for collection of data about the child from the mothers. Where mothers were unavailable, fathers completed the questionnaires. The questionnaire collected details on the child’s socio-demographic characteristics (age, sex, and socio-economic status), oral habits, and caries prevention habits. All study participants had oral examination performed on the same day.

### Malocclusion

Children were examined in their homes under natural light while sitting on a chair. The occlusal features of each child were assessed in the antero-posterior, transverse and vertical planes of space. The presence of individual malocclusion traits, i.e., crowding, spacing, increased overjet, reverse overjet, anterior open bite, increased overbite, buccal and lingual crossbite were documented. Malocclusion was assessed with the dental aesthetic index (DAI), described by Cons et al. [21] Scores for each of the 10 morphologic characteristics assessed by the DAI, i.e., number of missing visible teeth, crowding and spacing in the incisal segments, midline diastema, anterior irregularity in the maxillary and mandibular arches, anterior maxillary overjet and mandibular overjet, vertical anterior open bite and the antero-posterior molar relationships, were determined. The values obtained were multiplied with the appropriate weighting factor, summed, and added to a constant value of 13 to get the DAI score. The DAI scores were graded into four groups based on pre-defined DAI scores [21] 13–25, Grade 1 (normal or minor malocclusions, with slight or no treatment need); scores 26–30, Grade 2 (definite malocclusions, with treatment considered elective); scores 31–35, Grade 3 (severe malocclusions, with treatment highly desirable); and scores 36 and higher, Grade 4 (very severe or disabling malocclusions, with treatment considered mandatory).

### Caries

The caries status was assessed with the decayed, missing, and filled teeth/decayed, missing, and filled teeth (dmft/DMFT) index [22]. Caries severity was evaluated with the pufa/PUFA index [23]. Caries status was further divided into caries present or absent.

### Oral hygiene

Oral hygiene status of participants was evaluated with the simplified oral hygiene index (OHI-S) described by Greene and Vermillion [24]. The amount of debris or calculus present on the facial or lingual surfaces of six index teeth (A, E, F, K, O, and P) in the primary and 8, 3, 14, 19, 24, and 30 in the permanent dentition was used to determine the debris and calculus index scores, from which the OHI-S score was calculated. The oral hygiene was classified as good, fair, or poor when the scores were 0.0–1.2, 1.3–3.0, and > 3.0, respectively. Oral hygiene was further dichotomized into good oral hygiene and fair/poor oral hygiene.

### Gingival health

The presence and severity of gingivitis was evaluated with the gingival index, as described by Löe and Silness [25]. Changes in the gingiva in relation to the appropriate six index teeth (D, G, N, Q, K and T) in the primary and 7, 3, 12, 19, 23 and 28 in the permanent dentition were assessed. Four areas of each index tooth were scored, and the scores were summed and divided by four to give the gingival index for each tooth. The gingival index of each participant was obtained by adding the values of all index teeth and dividing by six. Gingivitis was classified as mild, moderate, or severe, with values of 0.1—1, 1.1—2, and 2.1–3, respectively. Gingivitis was dichotomized into mild gingivitis and moderate-to-severe gingivitis [26].

### Calibration of examiners

The five members of the research team responsible for data collection were calibrated before the study commenced to determine inter- and intra-examiner reproducibility. The mean κ coefficients obtained were 0.86 for caries, 0.92 for the OHI-S, 0.94 for gingival index, and 0.90 for malocclusion.

### Data analysis

The mean DAI scores of study participants were calculated. The association between the malocclusion traits, caries presence, oral hygiene status and gingival health were assessed with chi-square tests. Independent sample t-test was used for comparisons of the mean DAI scores. Malocclusion traits associated with the presence of caries, poor oral hygiene, and poor gingival health were also determined, using logistic regression. For the logistic regression model, oral hygiene status was dichotomized to good and poor (fair and poor) oral hygiene, and the severity of gingivitis was dichotomized to gingivitis present (moderate and severe) and gingivitis absent (mild). The Hosmer-Lemershow goodness-of-fit test was conducted to confirm the consistency of fit of the regression models. Also, collinearity was determined with tolerance and the VIF test. Statistical significance was inferred at *p* < 0.05.

### Ethical consideration

The Obafemi Awolowo University Teaching Hospital Complex Ile-Ife Ethics and Research Committee gave ethical approval for the study (ERC/2013/07/14). The Ife Central Local Government Authority also gave written approval to conduct the study. All the parents of study participants gave written informed consent for their children to participate; children aged eight to 12 years also provided written assent. Data collection was done without indicating the names of participants. Participants did not receive cash compensation for participating in the study.

## Results

The data of 992 children aged 1–12 years of the proposed 1011 study participants were complete enough for analysis of 98.1% of the proposed study participants. Incomplete data were excluded from the analysis. The data of 495 participants aged 6–12 years were extracted for this secondary analysis. The mean (SD) age of participants was 8.53 (1.90) years. Table 1 shows the age and sex distribution of the study participants. There were 242 (48.9%) male and 253 (51.1%) female participants, and most of the participants were 6–year-olds (20.2%).

**Table 1:** Age and sex profile of study participants (*N* = 495)

Age (years)	Male *N* (%)	Female *N* (%)	Total *N* (%)
6	49 (20.2%)	51 (20.2%)	100 (20.2%)
7	39 (16.1%)	33 (13.0%)	72 (14.5%)
8	48 (19.8%)	37 (14.6%)	85 (17.2%)
9	32 (13.2%)	36 (14.2%)	68 (13.7%)
10	35 (14.5%)	47 (18.6%)	82 (16.6%)
11	25 (10.3%)	26 (10.3%)	51 (10.3%)
12	14 (5.8%)	23 (9.1%)	37 (7.5%)
Total	242 (100%)	253 (100%)	495 (100%)

### Caries profile

The dmft scores ranged from 0 to 6 with a mean (SD) score of 0.27 (0.82). There were 125 unrestored carious teeth, four missing teeth due to caries, and three filled primary teeth. The DMFT scores ranged from 0 to 4 with a mean (SD) score of 0.07 (0.39). There were 28 unrestored carious teeth, three missing teeth, and two filled permanent teeth. The pufa score ranged from 0 to 4 with a mean (SD) score of 0.09 (0.43); study participants had 44 carious teeth with pulpal involvement, no tooth with ulceration, two teeth with fistula, and no tooth with abscess.

The PUFA score ranged from 0 to 3 with a mean (SD) score of 0.02 (0.20). Ten of the carious teeth had pulpal involvement, and one tooth had ulceration. No permanent tooth had fistula or was abscessed. Thirty-seven (7.5%) participants had pufa/PUFA score ≥ 1.

### Malocclusion profile

Table 2 presents the malocclusion and sex profiles of the study participants. The most common malocclusion traits were spacing, crowding and increased overjet, present in 297 (60%), 117 (23.6%), and 91 (18.4%) participants, respectively. Reverse overjet was observed in 24 (4.8%), increased overbite in 22 (4.4%), and anterior open bite in 21 (4.2%) participants. Only 12 (2.4%) and 9 (1.8%) participants had buccal and lingual cross bite, respectively. Among the male and female participants who had malocclusion, the only significant sex difference was with overjet: more male than female participants had increased overjet (21.9% vs 15.0%; *p* = 0.048).

**Table 2:** Malocclusion and sex profile of study participants (*N* = 495)

Variables	Male *N* (%) 242 (48.9%)	Female *N* (%) 253(51.1%)	Total *N* (%) 495(100%)	*p* value
Age				
6–12 years	242 (48.9%)	253 (51.1%)	495 (100%)	
Crowding				
Present	48 (19.8%)	69 (27.3%)	117 (23.6%)	0.052
Absent	194 (80.2%)	164 (72.7%)	378 (76.4%)	
Spacing				
Present	147(60.7%)	150 (59.3%)	297 (60.0%)	0.741
Absent	95 (39.3%)	103 (40.7%)	198 (40.0%)	
Increased overjet				
Present	53 (21.9%)	38 (15.0%)	91 (18.4%)	0.048
Absent	189 (78.1%)	215 (85.0%)	404 (81.6%)	
Reverse overjet				
Present	14 (5.8%)	10 (4.0%)	24(4.8%)	0.343
Absent	228 (94.2%)	243 (96.0%)	471(95.2%)	
Anterior open bite				
Present	12 (5.0%)	9 (3.6%)	21(4.2%)	0.439
Absent	230 (95.0%)	244 (96.4%)	474(95.8%)	
Increased overbite				
Present	7 (2.9%)	15 (5.9%)	22 (4.4%)	0.101
Absent	235 (97.1%)	238 (94.1%)	473(95.5%)	
Buccal crossbite				
Present	8 (3.3%)	4 (1.6%)	12 (2.4%)	0.209
Absent	234 (96.7%)	249 (98.4%)	483 (97.6%)	
Lingual crossbite				
Present	6 (2.5%)	3 (1.2%)	9 (1.8%)	0.278
Absent	236 (97.5%)	250 (98.8%)	486 (98.2%)	

Participants’ DAI scores ranged from 13 to 48 with a mean (SD) score of 20.7 (4.57). Four hundred and fifty (90.9%) participants had no need for orthodontic treatment. Treatment need was elective for 26 (5.3%), highly desirable for 11 (2.2%) and mandatory for 8 (1.6%) participants.

### Oral hygiene profile

The mean (SD) OHI-S score was 1.56 (0.74). One hundred and ninety-five (39.4%) children had good oral hygiene; 278 (56.2%) had fair oral hygiene; and only 22 (4.4%) had poor oral hygiene. There was no significant difference between the mean OHI-S scores of male and female participants (1.62 vs 1.51; *p* = 0.086).

### Gingival health profile

The mean (SD) gingival index score was 0.90 (0.43). Three hundred and sixty-one (72.9%) study participants had mild gingivitis, 133 (26.9%) had moderate gingivitis, and one (0.2%) had severe gingivitis. No significant gender difference was observed in the mean gingival index scores (0.92 vs 0.89; *p* = 0.56). Of the 37 (7.5%) participants with pufa/PUFA score ≥ 1, 20 (54.1%) had mild gingivitis, 16 (43.2%) had moderate gingivitis, and one (2.7%) had severe gingivitis.

### Malocclusion and caries

Table 3 highlights the malocclusion and caries profile of study participants. Significant differences were observed in the proportions that had crowding (33.8% vs 21.9%; *p* = 0.026) and had buccal crossbite (6.8% vs 1.7%; *p* = 0.009) and caries. There were no significant differences in the proportion of participants with and without spacing, increased overjet, reverse overjet, anterior open bite, increased overbite, and lingual crossbite who had caries.

**Table 3:** Malocclusion, caries, oral hygiene and gingivitis profile of study participants (*N* = 495)

Variables	Caries present *n* = 74	Caries absent *n* = 421	*P* value	Good oral hygiene *N* = 195 *N* (%)	Fair/poor oral hygiene *N* = 300 *N* (%)	*P* value	Mild gingivitis *N* = 361 *N* (%)	Moderate/Severe gingivitis *N* = 134 *N* (%)	*P* value	Total *N* = 495 *N* (%)
Age										
6–12 years	74 (14.9%)	421 (85.1%)		195 (39.4%)	300 (60.6%)		361 (72.9%)	134 (27.1%)		495 (100%)
Sex										
Male	30 (40.5%)	212 (50.4%)	0.119	89 (45.6%)	153 (51.0%)	0.244	175 (48.5%)	67 (50.0%)	0.763	242 (48.9%)
Female	44 (59.5%)	209 (49.6%)		106 (54.4%)	147 (49.0%)		186 (51.5%)	67 (50.0%)		253 (51.1%)
Crowding										
Present	25 (33.8%)	92 (21.9%)	0.026	50 (25.6%)	67 (22.3%)	0.397	93 (25.8%)	24 (17.9%)	0.068	117 (12.6%)
Absent	49 (66.2%)	329 (78.1%)		145 (74.4%)	233 (77.7%)		268 (74.2%)	110 (82.1%)		378 (76.4%)
Spacing										
Present	45 (60.8%)	252 (59.9%)	0.877	119 (61.0%)	178 (59.3%)	0.707	210 (58.2%)	87 (64.9%)	0.173	297 (60.0%)
Absent	29 (39.2%)	169 (40.1%)		76 (39.0%)	122 (40.7%)		151 (41.8%)	47 (35.1%)		198 (40.0%)
Increased overjet										
Present	14 (18.9%)	77 (18.3%)	0.897	37 (19.0%)	54 (18.0%)	0.785	55 (15.2%)	36 (26.9%)	0.003	91 (18.4%)
Absent	60 (81.1%)	344 (81.7%)		158 (81.0%)	246 (82.0%)		306 (84.8%)	98 (73.1%)		404 (81.6%)
Reverse overjet										
Present	3 (4.1%)	21 (5.0%)	0.724	9 (4.6%)	15 (5.0%)	0.846	20 (5.5%)	4 (3.0%)	0.218	24 (4.8%)
Absent	71 (95.9%)	400 (95.0%)		186 (95.4%)	285 (95.0%)		341 (94.5%)	130 (97.0%)		471 (95.2%)
Anterior open bite										
Present	2 (2.7%)	19 (4.5%)	0.451	8 (4.1%)	13 (4.3%)	0.901	10 (2.8%)	11 (8.2%)	0.008	21 (4.2%)
Absent	72 (97.3%)	402 (95.5%)		187 (95.9%)	287 (95.7%)		351 (97.2%)	123 (91.8%)		474 (95.8%)
Increased overbite										
Present	2 (2.7%)	20 (4.8%)	0.402	7 (3.6%)	15 (5.0%)	0.457	19 (5.3%)	3 (2.2%)	0.121	22 (4.4%)
Absent	72 (97.3%)	401 (95.2%)		188 (96.4%)	263(95.0%)		342 (94.7%)	131 (97.8%)		473 (95.6%)
Buccal crossbite										
Present	5 (6.8%)	7 (1.7%)	0.009	3 (1.5%)	9 (3.0%)	0.287	9 (2.5%)	3 (2.2%)	0.869	12 (2.4%)
Absent	69 (93.2%)	414 (98.3%)		192 (98.5%)	291 (97.0%)		352 (97.5%)	131 (97.8%)		483 (97.6%)
Lingual crossbite										
Present	2 (2.7%)	7 (1.7%)	0.559	1 (0.5%)	8 (2.7%)	0.159	8 (2.2%)	1 (0.7%)	0.236	9 (1.8%)
Absent	72 (97.3%)	414 (98.3%)		194 (99.5%)	292 (97.3%)		353 (97.8%)	133 (99.3%)		486 (98.2%)

Most of the study participants with dmft/DMFT ≥1 [dmft (95.7%) and DMFT (91.3%)] had no need for orthodontic treatment. Only four (4.4%) participants with dmft/DMFT ≥1 had need for orthodontic treatment, which ranged from elective to mandatory treatment.

### Malocclusion and Oral hygiene status

Table 3 also highlights the oral hygiene status of the study participants. There were no significant differences in the oral hygiene status of participants with and without malocclusion traits.

### Malocclusion and gingivitis

The relationship between malocclusion traits and gingivitis is also highlighted in Table 3. A greater proportion of participants with increased overjet had moderate to severe gingivitis than had mild gingivitis (26.9% vs 15.2%; *p* = 0.003). Also, a greater proportion of participants with anterior open bite had moderate to severe gingivitis than had mild gingivitis (8.2% vs 2.8%; *p* = 0.008). There were no significant differences in the proportion of participants with and without crowding, spacing, reverse overjet, increased overbite buccal and lingual crossbite and the gingival health status.

### Malocclusion, caries, Oral hygiene status, and gingival health status

Table 4 shows the association between malocclusion, assessed according to DAI score, and caries, oral hygiene and gingival health status. The mean DAI scores of participants with mild gingivitis compared with moderate/severe gingivitis differed significantly (20.21 vs 22.11; *p* = 0.001). There was no significant difference in the mean DAI scores of participants with and without caries (21.0 vs 20.77; *p* = 0.507), and the DAI scores of participants with good oral hygiene compared with those with fair/poor oral hygiene. (21.0 vs 20.56; *p* = 0.349).

**Table 4:** Comparisons of mean DAI scores of study participants (*N* = 495)

Variables	Mean DAI score	SD	*p* value
Dental caries			
Caries present (*n* = 74)	21.04	5.25	0.507
Caries absent (*n* = 421)	20.66	4.44	
Oral hygiene			
Good oral hygiene (*n* = 195)	20.95	4.24	0.349
Fair/Poor oral hygiene (*n* = 300)	20.56	4.76	
Gingivitis			
Mild gingivitis (*n* = 361)	20.21	3.83	0.001
Moderate/Severe gingivitis (*n* = 134)	22.06	5.92	

### Malocclusion traits associated with caries

Table 5 highlights the results of the logistic regression analysis to determine the malocclusion traits associated with the presence of caries. The Hosmer-Lemershow goodness-of-fit test confirmed the consistency of fit of the model (*p* = 0.078). Collinearity statistics showed that tolerance was > 0.10, while VIF was < 10. None of the variables suffered from multicollinearity. The presence of poor oral hygiene, crowding, and buccal crossbite were associated with the presence of caries. Poor oral hygiene (OR: 1.83; CI: 1.05–3.18; *p* = 0.033), crowding (OR: 1.97; CI: 1.01–3.49; *p* = 0.021), and buccal crossbite (OR: 6.57; CI: 1.51–28.51; *p* = 0.012) significantly increased the odds of having caries.

**Table 5:** Results of Logistic Regression Analysis for the association between variables and presence of caries and gingivitis in a Sample of 495 Children

Demographic variables	Caries		Gingivitis	
	Multivariate regression		Multivariate regression	
	Odds Ratio (95% CI)	*P* value	Odds Ratio (95% CI)	*P* value
Age	1.09 (0.95–1.25)	0.218	1.10 (0.97–1.21)	0.142
Gender (Female)	1.61 (0.95–2.70)	0.079	0.93 (0.61–1.54)	0.750
Oral hygiene	1.83 (1.05–3.18)	0.033	0.44 (0.28–0.70)	< 0.001
Crowding	1.97 (1.01–3.49)	0.021	1.50 (0.87–2.58)	0.138
Spacing	1.27 (0.73–3.49)	0.400	0.84 (0.54–1.31)	0.446
Increased Overjet	1.20 (0.63–2.31)	0.578	0.46 (0.28–0.77)	0.003
Reverse Overjet	0.75 (0.21–2.68)	0.754	1.88 (0.58–6.14)	0.295
Anterior Open Bite	0.53 (0.12–2.41)	0.407	0.31 (0.12–0.79)	0.014
Increased Overbite	0.45 (0.10–2.03)	0.298	3.32(0.92–11.98)	0.066
Buccal Crossbite	6.57 (1.51–28.51)	0.012	0.75 (0.15–3.78)	0.729
Lingual Crossbite	0.46 (0.06–3.77)	0.471	3.70(0.36–37.69)	0.269
Constant	1.58	0.783	0.07	0.093

### Malocclusion traits associated with gingivitis

Table 5 also highlights the results of the logistic regression to determine the traits associated with gingivitis. The Hosmer-Lemershow goodness-of-fit test confirmed the consistency of fit of the model (*p* = 0.238). Collinearity statistics showed that tolerance was > 0.10, while VIF was < 10. None of the variables suffered from multicollinearity. Poor oral hygiene (*p* < 0.001), increased overjet (*p* = 0.003), and anterior open bite (*p* = 0.014) were the only significant traits associated with gingivitis.

## Discussion

The results of the study indicate that for children aged 6 to 12 years, crowding and buccal cross bite were associated with caries, and increased overjet and anterior open bite were associated with moderate/severe gingivitis. These finding indicate the need to give priority to children with these malocclusion traits for treatment.

The study findings contribute to the debate on the justification for recommending orthodontic treatment to improve oral health in view of conflicting data on the effect of malocclusion on oral health. Like Ngom et al. [11] had opined, the study findings suggest that providing orthodontic treatment reduces the risk for caries and gingivitis in young children. Although suggestions that certain malocclusion traits call only for special professional efforts of oral hygiene education, rather than orthodontic therapy [10], we found that the oral hygiene need of the study population was not limited to those who had malocclusion. Malocclusion may therefore have other direct and or indirect pathways of association with caries and gingivitis beyond oral hygiene practices. There is no conceptual framework that defines these potential pathways, however. More studies are needed to understand how malocclusion predisposes to oral health problems.

While prior studies have identified an association between crowding and dental caries due to food accumulation and plaque retention in areas of crowding [27–29], we identified an association between crowding and caries like other studies, and between buccal crossbite and caries, which has not been described before now. Children with buccal crossbite have the buccal cusps of some posterior upper teeth positioned buccal to the lower teeth in centric occlusion. Buccal crossbite may increase the difficulty of cleaning the teeth in both arches, and some teeth could also become non-functional, thereby increasing plaque retention [30] and caries development. We did notice that our confidence interval was wide, largely due to the small number of participants with this trait. Tooth spacing could also be a plaque retention factor and increase the risk for caries [31], although we found no such association in this study.

The malocclusion traits associated with gingivitis in this study- increased overjet and anterior open bite - may result from increased plaque accumulation due to mouth breathing [32] and difficulty with tooth cleaning [33]. Prior studies that identified an association between malocclusion and gingivitis [9, 10] reported that periodontal disease was significantly more frequent in the maxilla in children with extreme maxillary overjet. Increased overjet and anterior open bite are closely associated with lip incompetence, hyperplastic gingivitis around the upper incisors, and gingivitis due to drying out of the oral mucosa in the absence of lip cover and the cleansing effect of saliva [34]. Also, the presence of nonfunctional teeth in children with anterior open bite contributes to plaque and debris accumulation and gingivitis.

Unlike studies that identified an association between crowding and gingivitis [35–38], we found no association between the two features. Our finding may be an age-dependent phenomenon: individual host factor is the risk potential for chronic inflammatory processes whose consequences manifest at an older age [32]. An association between crowding and gingivitis may therefore be age-dependent, with the risk higher in older adolescents and adults.

Our study has a few limitations. First, it is a cross-sectional study, so we could not establish cause-effect relationships. Second, it is a secondary data analysis, so it was not powered to determine the association between malocclusion, caries and gingivitis. Third, the study did not identify the locations of the malocclusion traits and associate them with the sites of caries and gingivitis. Despite these limitations, the study provides evidence suggestive that malocclusion has a deleterious effect on the oral health for children in the mix dentition period.

## Conclusions

In this study on the association between malocclusion, caries and oral hygiene in children 6 to 12 years old resident in suburban Nigeria, we found that crowding and buccal crossbite were associated with caries, whereas increased overjet and anterior open bite were associated with gingivitis. Gingivitis was also associated with the severity of malocclusion. These findings justify the recommendation of orthodontic treatment with the aim of improving oral health.

## Data Availability

The datasets generated used and/or analyzed during the current study are available from the corresponding author on reasonable request.

## Abbreviations

DAI: Dental Aesthetic Index
dmft/DMFT: Decayed, Missing, and Filled Teeth index
OHI-S: Simplified Oral Hygiene Index
pufa/PUFA: Pulpal Involvement, Ulceration Fistula and Abscess Index
